# Probiotic Ferulic Acid Esterase Active *Lactobacillus fermentum* NCIMB 5221 APA Microcapsules for Oral Delivery: Preparation and *in Vitro* Characterization

**DOI:** 10.3390/ph5020236

**Published:** 2012-02-16

**Authors:** Catherine Tomaro-Duchesneau, Shyamali Saha, Meenakshi Malhotra, Michael Coussa-Charley, Imen Kahouli, Mitchell L. Jones, Alain Labbé, Satya Prakash

**Affiliations:** 1 Biomedical Technology and Cell Therapy Research Laboratory, Departments of Biomedical Engineering, Physiology, and Artificial Cells and Organs Research Center, Faculty of Medicine, McGill University, 3775 University Street, Montreal, Quebec, H3A 2B4, Canada; Email: catherine.tomaro-duchesneau@mail.mcgill.ca (C.T.-D.); meenakshi.malhotra@mail.mcgill.ca (M.M.); michael.coussa-charley@mail.mcgill.ca (M.C.-C.); 2 Faculty of Dentistry, McGill University, 3775 University Street, Montreal, Quebec, H3A 2B4, Canada; Email: shyamali.saha@mail.mcgill.ca; 3 Department of Experimental Medicine, McGill University, 3775 University Street, Montreal, Quebec, H3A 2B4, Canada; Email: imen.kahouli@mail.mcgill.ca; 4 Micropharma Limited, 141 President Kennedy Ave., UQAM Biological Sciences Building, 5th Floor, Suite 5569, Montreal, Quebec, H2X 3Y7, Canada; Email: mitchell@micropharma.net (M.L.J.); alain@micropharma.net (A.L.)

**Keywords:** *Lactobacillus fermentum*, artificial cells, ferulic acid esterase, microcapsules

## Abstract

Probiotics possess potential therapeutic and preventative effects for various diseases and metabolic disorders. One important limitation for the oral delivery of probiotics is the harsh conditions of the upper gastrointestinal tract (GIT) which challenge bacterial viability and activity. One proposed method to surpass this obstacle is the use of microencapsulation to improve the delivery of bacterial cells to the lower GIT. The aim of this study is to use alginate-poly-L-lysine-alginate (APA) microcapsules to encapsulate *Lactobacillus fermentum* NCIMB 5221 and characterize its enzymatic activity and viability through a simulated GIT. This specific strain, in previous research, was characterized for its inherent ferulic acid esterase (FAE) activity which could prove beneficial in the development of a therapeutic for the treatment and prevention of cancers and metabolic disorders. Our findings demonstrate that the APA microcapsule does not slow the mass transfer of substrate into and that of the FA product out of the microcapsule, while also not impairing bacterial cell viability. The use of simulated gastrointestinal conditions led to a significant 2.5 log difference in viability between the free (1.10 × 10^4^ ± 1.00 × 10^3^ cfu/mL) and the microencapsulated (5.50 × 10^6^ ± 1.00 × 10^5^ cfu/mL) *L. fermentum* NCIMB 5221 following exposure. The work presented here suggests that APA microencapsulation can be used as an effective oral delivery method for *L. fermentum* NCIMB 5221, a FAE-active probiotic strain*.*

## 1. Introduction

**Figure 1:**
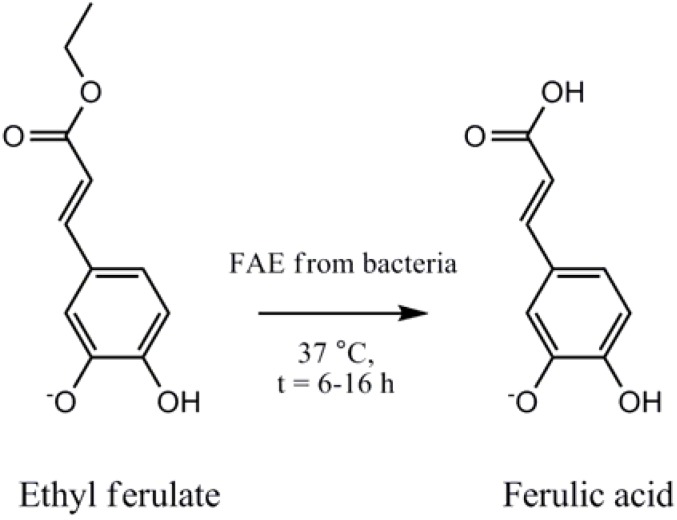
Ferulic acid esterases (FAE) enable microbes to hydrolyse the ester bond between hydroxyl cinnamic acids and sugars. The hydrolysis of ethyl ferulate by FAE gives rise to ferulic acid a compound with a number of health-promoting benefits.

Ferulic acid (FA), a naturally found phenolic acid, is a potent antioxidant able to neutralize free radicals, such as Reactive Oxygen Species (ROS) [1]. ROS have been implicated in DNA damage, cancer and accelerated cell aging [2]. Recent studies suggest that FA has antitumor activity against breast cancer [3,4] liver cancer [5,6] and is effective at preventing cancer induced by the exposure to various carcinogenic compounds such as benzopyrene [7] and 4-nitroquinoline 1-oxide [8]. The oral delivery of free FA is hampered by its quick absorption in the small intestine, specifically in the jejunum, followed by its rapid excretion [9,10]. However, it has been proposed, and shown in previous studies that some GIT bacterial strains produce FAE, an enzyme that has the inherent capability to produce FA from available substrates in the GIT (see Figure 1).

The oral delivery of conjugated FA, in the form of a dietary source such as wheat bran, is a feasible alternative, with the release of free FA by microbial FAE present in the lower human digestive tract, giving rise to a constant and controlled release of FA [9,10]. The development of probiotic formulations to enhance the FA bioavailability should prove beneficial for the treatment and prevention of inflammatory metabolic disorders [11].Previous research by our group has demonstrated the use of *Lactobacillus fermentum* NCIMB 5221 as a superior microbial producer of FA [12]. The oral delivery of probiotics, however, is impeded by the harsh conditions of the upper GIT, specifically the presence of bile and a detrimentally acidic pH [13]. Microencapsulation, a method defined as the “entrapment of a compound or a system inside a dispersed material for its immobilization, protection, controlled release, structuration and functionalisation” has been used to overcome the challenge of delivering bacterial cells through the GIT [14]. This article investigates the use of APA microencapsulation for the viable delivery of the FAE active *Lactobacillus fermentum* NCIMB 5221 to the colon. The results should demonstrate the efficiency of APA microcapsules to increase the viability of this specific probiotic strain in the GIT while preserving enzymatic activity in terms of FA production.

## 2. Experimental Methods and Materials

### 2.1. Bacterial Growth Media and Chemicals

Ethyl ferulate (ethyl 4-hydroxy-3-methoxycinnamate, EFA) and ferulic acid (*trans*-4-hydroxy-3-methoxycinnamate, FA) were purchased from Sigma-Aldrich (Oakville, ON, Canada). De Man, Rogosa, Sharpe (MRS) broth and Methanol of high-performance liquid chromatography (HPLC) grade were obtained from Fisher Scientific (Ottawa, ON, Canada). Water was purified with an EasyPure reverse osmosis system and a NanoPure Diamond Life Science (UV/UF) ultrapure water system from Barnstead (Dubuque, IA, USA). All other chemicals were of analytical or HPLC grade and purchased from commercial sources.

### 2.2. Bacterial Strains and Culture Conditions

*Lactobacillus fermentum* NCIMB 5221 was purchased from NCIMB (Aberdeen, Scotland, UK). The bacterial strain was stored at −80 °C in MRS containing 20% (v/v) glycerol. An MRS-agar plate was streaked for isolation from the frozen stock and incubated at 37 °C with 5% CO_2_ for 24 h to ensure purity. One colony from the MRS-agar plate was inoculated into 5 mL of MRS broth and incubated at 37 °C for 24 h. A 1% (v/v) inoculum was then used for subculturing and incubated at 37 °C for 24 h immediately before use.

### 2.3. APA Microencapsulation

The microencapsulation of *L. fermentum* NCIMB 5221 was performed according to the standard protocol, with slight modifications to the flow rate, vibration frequency and voltage [15]. The microcapsules were loaded with 8% (w/v) bacterial pellet. A sodium-alginate solution (1.75% w/v) containing the *L. fermentum* NCIMB 5221 was extruded into a stirred CaCl_2_ solution (0.1 M) using a microencapsulator and a 200 μm nozzle (Inotech Corp.). The formed calcium-alginate beads were washed in a physiological solution (PS) followed by their immersion in a poly-L-lysine (PLL) solution (0.1% w/v) for 20 min. Another wash in PS was performed for 5 min followed by immersion in a sodium-alginate solution (0.1% w/v) for 20 min. The resulting microcapsules were stored in minimal storage media at 4 °C for further testing. Viability on microcapsules was performed by exposure to 0.1 M sodium citrate until disruption of the microcapsule was observed. Ten-fold serial dilutions in physiological saline followed by plating on MRS-agar plates were then performed to determine the colony forming units.

### 2.4. Ferulic Acid Esterase HPLC Assay To Measure FA Production

The bacterial strain was subcultured from MRS broth at 1% (v/v) to MRS-EFA broth at an EFA concentration of 1.33 mM (0.2956 mg/mL). For the APA microcapsules these were added at 10% (w/v) into MRS-EFA broth. Uninoculated MRS-EFA broth was used as a negative control and treated in the exact same way. Each sample was treated in triplicate and incubated at 37 °C during the course of the experiment. An HPLC assay, modified from Mastihuba *et al.*, was used to measure FAE activity [16]. At each time point, 500 μL of sample was added to tubes and centrifuged at 10,000 rpm for 7 min at 4 °C. The resulting supernatant (300 μL) was acidified with 0.35 M H_2_SO_4 _(100 μL) and vortexed. 1mM benzoic acid (300 μL) was added, as an internal standard, followed by the addition of 0.7 M NaOH (100 μL) to neutralize the pH. The samples were then stored at −20 °C until all of the samples were collected for the HPLC analysis.

For HPLC analysis, the samples were thawed to room temperature and filtered with a 0.45 μm filter. The analysis was performed on a reverse-phase C-18 column: LiChrosorb RP-18, 25 × 0.46 cm (Grace Davison Discovery Sciences, ON, Canada). The HPLC system consists of a ProStar 335 diode array detector (DAD) set at 280 nm and 320 nm, a ProStar 410 autosampler, and the software Star LC workstation version 6.41. 25 μL was injected for each sample. The mobile phase (solvent A) consists of 37% (v/v) methanol and 0.9% (v/v) acetic acid in water (HPLC grade). Solvent B consisted of 100% (v/v) methanol. The run was initiated with solvent A at 100% for 16 minutes. This was followed by a 1 minute linear gradient to reach 100% of solvent B, attained at the 17th minute. Solvent B was isocratically held at 100% for 12 minutes, until the 29th minute. This was followed by a 1 minute linear gradient to reach 100% of solvent A by the 30th minute. Standard curves of FA and EFA, using peak area quantification, were generated for quantifying the test sample FA and EFA concentrations. The FA standard curve was generated using triplicates and the concentrations 100, 300, 500, 960 and 1,100 μM were plotted against peak area (R^2^ = 0.9869). The EFA standard curve was generated using triplicates and the concentrations 100, 300, 500, 700, 1,000, 1,400 and 1,800 μM were plotted against peak area (R^2^ = 0.9785). Standards and quality control samples were prepared and analyzed in the same way as the test samples.

### 2.5. Simulated Gastrointestinal Conditions to Determine the Stability and Viability of Microencapsulated L. Fermentum NCIMB 5221 Delivered Orally

Simulated gastric (SGF) and intestinal fluids (SIF) were prepared according to the U.S. Pharmacopeia, with some minor modifications [17]. SGF consisted of NaCl (2 g/L), glucose (3.5 g/L) and pepsin (3.2 g/L) in deionized water. The pH of the SGF was adjusted to 1.5 using 2 M HCl. SIF consisted of monobasic potassium phosphate (6.8 g/L), pancreatin (10 g/L), Oxgall (1.5 g/L) and glucose (3.5 g/L) in deionized water. 20 g of APA microcapsules or 2 g of free *L. fermentum* NCIMB 5221 were added to 200 mL SGF and incubated at 37 °C on a rotary shaker at 75 rpm for 2 h. Following the 2 h, 200 mL of SIF was added into each flask and the pH was increased to 6.8 using 2 M NaOH. At each time point, 1 mL of the solution was sampled into 9 mL of 0.1 M sodium citrate. This was serially diluted in 10-fold dilutions in 0.85% (w/v) NaCl and plated on MRS agar in triplicates. These MRS-agar plates were incubated at 37 °C and 5% CO_2_, followed by colony counting following 48 h of incubation.

### 2.6. Statistical Analysis

Values are expressed as means ± Standard Deviation. Statistical analysis was carried out using Minitab (Minitab, Version 14, Minitab Inc., State College, PA, USA). Statistical comparisons between EFA/FA concentrations were carried out by using the general linear model (GLM) and post-hoc analysis. Statistical significance was set at *p* < 0.05. All interaction terms were treated as fixed terms and *p*-values less than 0.01 were considered highly significant.

## 3. Results and Discussion

### 3.1. Results

**Figure 2:**
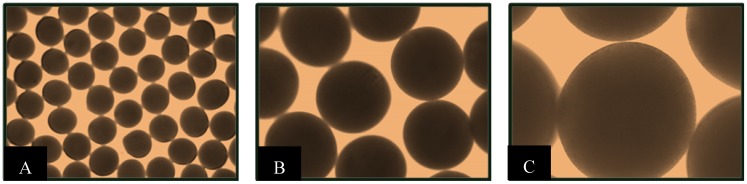
Morphology of APA microcapsule containing *L. fermentum* NCIMB 5221 taken by light microscope (**A**) 40× (**B**) 100× (**C**) 200×. The approximate diameter of the microcapsules was 400 ± 25 μm.

*L. fermentum* NCIMB 5221 microcapsules were formed to investigate the suitability of the APA microcapsule for the oral delivery of an FAE active probiotic. *L. fermentum* NCIMB 5221 microencapsulation was optimized—by controlling the flow rate, stirring time, stirring speed, coating time, vibration frequency, and voltage—to obtain monodispersed and spherical microcapsules. The obtained microcapsules were observed under light microscopy at magnifications of 40×, 100× and 200×. The APA microcapsules obtained were monodispersed, as seen in Figure 2A, with an approximate size of 400 ± 25 μm in diameter. Figure 2B demonstrates the spherical shape of the microcapsules and Figure 2C allows for visualisation of the outer coat of the APA microcapsule loaded with *L. fermentum* NCIMB 5221 at 8% of the total microcapsule weight. The viability of the bacteria prior to microencapsulation, in the alginate solution, was determined to be 1.95 × 10^9^ ± 2.1 × 10^8^ cfu/mL and the microcapsules had a final viability of 1.21 × 10^9^ ± 9.54 × 10^7^ cfu/g, suggesting no significant loss of bacterial viability due to the process of APA microencapsulation.

**Figure 3:**
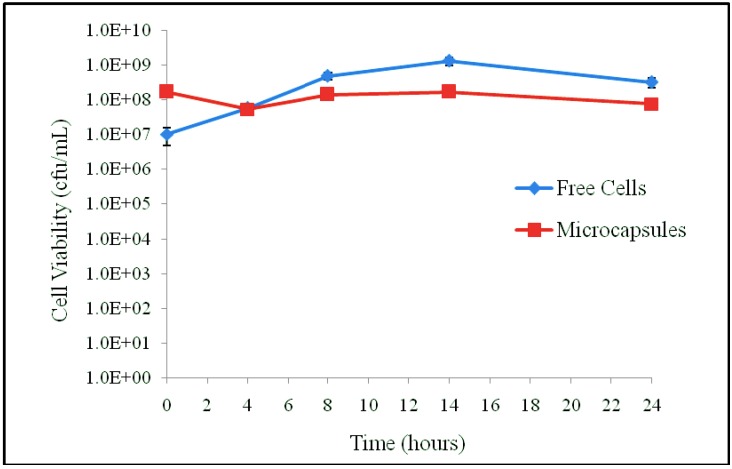
The viability of the free and microencapsulated *L. fermentum* NCIMB 5221 during the FAE assay (MRS-EFA 0.2956 mg/mL at 37 °C). Each point represents the mean of triplicates and the error bars the standard deviation. During the assay, there was no significant difference in viability between the free and microencapsulated bacteria.

The FAE activity of APA microencapsulated *L. fermentum* NCIMB 5221 was then determined to ensure that the APA microcapsule does not impede the uptake of the substrate, EFA, and the release of the desired product, FA. For this, the viability of free and microencapsulated *L. fermentum* NCIMB 5221 during the FAE assay was determined, as can be observed in Figure 3.

At the start of the assay, the free cells had a viability of 1.53 × 10^9^ ± 9.02 × 10^7^ cfu/mL and the microcapsules a viability of 6.77 × 10^8^ ± 7.77 × 10^7^ cfu/mL. Following 24 h of incubation, the free cells had a viability of 3.27 × 10^8^ ± 1.00 × 10^3^ cfu/mL and the microcapsules a viability of 7.72 × 10^7^ ± 1.00 × 10^5^ cfu/mL.

**Figure 4:**
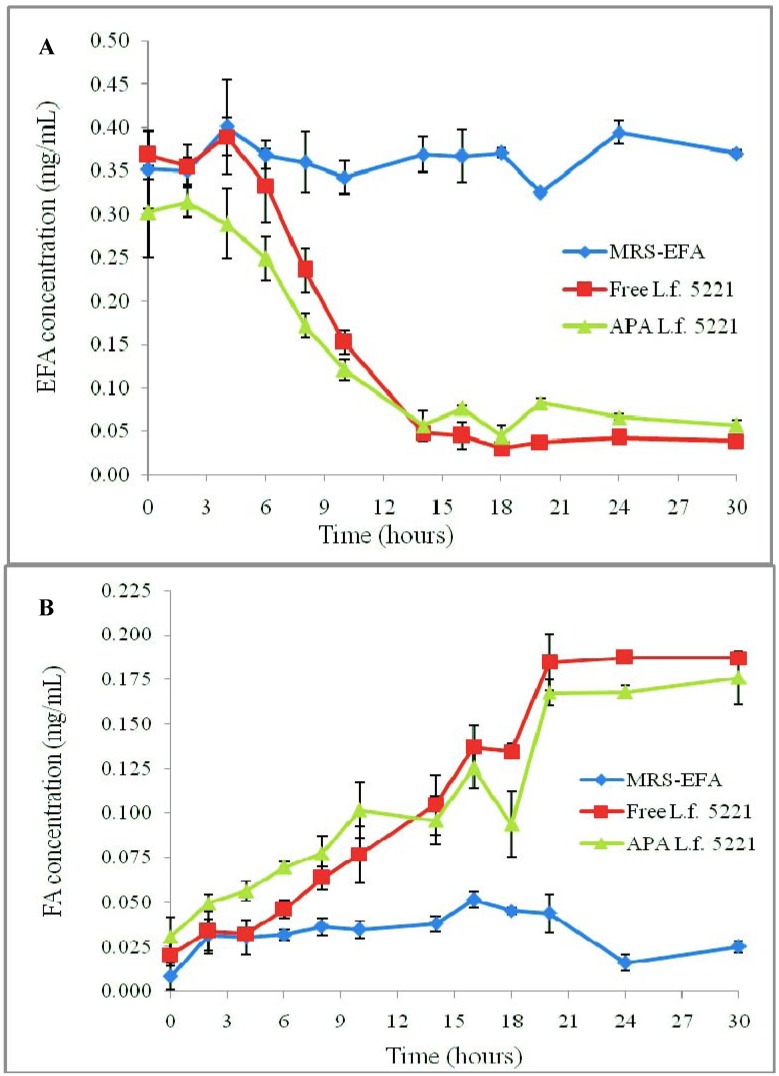
FAE quantitative HPLC assay of free (free L.f. 5221) and microencapsulated *L. fermentum* NCIMB 5221 (APA L.f. 5221). Uninoculated MRS-EFA was used as a negative control. The presented data represents the amount of unhydrolysed EFA remaining in solution (**A**) and the amount of FA produced (**B**), as measured by HPLC peak area data. Each point represents the mean of triplicates and the error bars represent the standard deviations.These results demonstrate no significant difference in FA production between the free and encapsulated *L. fermentum* NCIMB 5221.

In terms of FAE activity, the initial EFA concentration was 0.2956 mg/mL. Following 30 h of incubation, the free cells had 0.0382 ± 0.0011 mg/mL (12.92 ± 0.37%) and the microcapsules had 0.057 ± 0.0054 mg/mL (19.28 ± 1.83%) EFA remaining in solution, as seen in Figure 4A. At this point, the free cells had a total production of 0.1872 ± 0.0033 mg/mL FA and APA microcapsules had a final production of 0.1760 ± 0.0149 mg/mL FA, as seen in Figure 4B.

**Figure 5:**
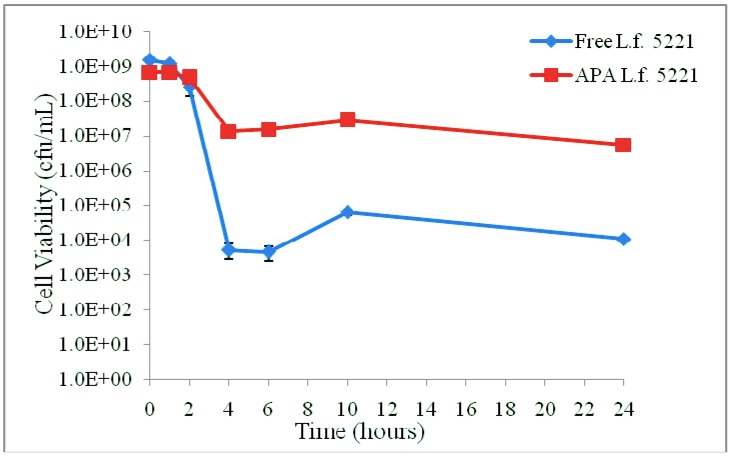
The viability of *L. fermentum* NCIMB 5221 in free and encapsulated form was determined upon exposure to the simulated GI conditions using standard colony counting methods. Each point represents the mean of triplicates and the error bars the standard deviations. A significant difference in viability between free and microencapsulated bacterial cells following simulated GI exposure was observed (*p* < 0.001; Tukey’s HSD).

The viability of *L. fermentum* NCIMB 5221 in free and encapsulated form was determined upon exposure to the simulated gastrointestinal conditions, Figure 5. The initial viability of *L. fermentum* NCIMB 5221 was 1.53 × 10^9^ ± 9.02 × 10^7^ cfu/mL for the free cells and 6.77 × 10^8^ ± 7.77 × 10^7^ cfu/mL for the microencapsulated cells. Following 2 h of exposure to the simulated conditions of the stomach the viability was 2.60 × 10^8^ ± 1.22 × 10^8^ cfu/mL for the free cells and 4.73 × 10^8^ ± 4.93 × 10^7^ cfu/mL for the encapsulated cells. Following the further exposure to simulated intestinal conditions for 24 h, the free *L. fermentum* NCIMB 5221 demonstrated a viability of 1.10 × 10^4^ ± 1.00 × 10^3^ cfu/mL and the microencapsulated *L. fermentum* NCIMB 5221 had a viability of 5.50 × 10^6^ ± 1.00 × 10^5^ cfu/mL. The viability of *L. fermentum* NCIMB 5221, through the *in vitro* gastrointestinal passage, and the associated conditions of the gastric and intestinal exposure, are summarized in Table 1. It is also noted that, following the transition to the intestinal conditions, the microcapsules lost some of their integrity due to a change in pH and osmotic conditions.

**Table 1:** The viability of *L. fermentum* NCIMB 5221, through the *in vitro* GI passage and the associated conditions of the gastric and intestinal exposure. Free = free *L. fermentum* NCIMB 5221; APA = microencapsulated *L. fermentum* NCIMB 5221. The data values represent the mean of triplicates ± SD. These results demonstrate a significant difference in viability between the free and microencapsulated cells (*p* < 0.001; Tukey’s HSD).

Time (hours)	GIT section	pH	Solutes	Viability (cfu/mL)		Viability (%)	
				Free	APA	Free	APA
0	Stomach	1.5	Sodium chloride Peptic enzymes Glucose	1.53 × 10^9^ ± 9.02 × 10^7^	6.77 × 10^8^ ± 7.77 × 10^7^	100.00 ± 0.059	100.00 ± 0.115
1				1.18 × 10^9^ ± 2.04 × 10^8^	6.83 × 10^8^ ± 4.51 × 10^7^	77.51 ± 13.365	100.99 ± 6.664
2				2.60 × 10^8^ ± 1.22 × 10^8^	4.73 × 10^8^ ± 4.93 × 10^7^	17.03 ± 7.969	69.95 ± 7.290
4	Small / Large intestines	6.8	Potassium phosphate Pancreatic enzymes Bile Glucose	5.33 × 10^3^ ± 2.52 × 10^3^	1.35 × 10^7^ ± 1.12 × 10^6^	0.0004 ± 0.0002	2.00 ± 0.165
6				4.67 × 10^3^ ± 2.08 × 10^3^	1.53 × 10^7^ ± 1.15 × 10^6^	0.0003 ± 0.0001	2.26 ± 0.170
10				6.37 × 10^4^ ± 1.33 × 10^4^	2.82 × 10^7^ ± 1.23 × 10^6^	0.0042 ± 0.0009	4.17 ± 0.182
24				1.10 × 10^4^ ± 1.00 × 10^3^	5.50 × 10^6^ ± 1.00 × 10^5^	0.0007 ± 0.0001	0.813 ± 0.015

### 3.2. Discussion

Past and recent research has looked at probiotics for use as therapeutics. These formulations are defined as dietary supplements containing bacteria which, when administered in adequate amounts, confer a health benefit on the host [13,18,19]. Probiotics, as natural compounds, are generally considered safe, but can also be tested for set-out safety parameters [20]. A number of studies have investigated bacterial strains for a range of conditions, including infections, allergies and metabolic disorders such as ulcerative colitis and Crohn’s disease [21,22,23,24,25]. Promising research focuses on the microbial secretion and production of beneficial biologically active enzymes and proteins [26,27]. These include the use of ornithine decarboxylase as a powerful antioxidant for the treatment of autoimmune diseases and accelerated cell apoptosis [28], the use of bile salt hydrolase for hypercholesterolemia [29,30,31], and the use of bile transport and tolerance proteins for the efficient delivery of probiotics [32]. In recent studies, the products of, another microbial protein, cinnamoyl esterase, have shown significant levels of antioxidant activity [12,33] and other effects, including stimulation of insulin secretion [34,35], prevention of oxidative stress [33], lipid peroxidation [36], cholesterol-lowering capabilities [11] and inhibition of diabetic nephropathy progression [37]. FA, a well-characterised antioxidant, is one of the desired products of hydrolysis by FAE [12].

Previously we have screened strains for FAE activity and selected *L. fermentum* NCIMB 5221 as the best FA producer of the investigated Lactobacilli strains [12]. For the development of an efficient probiotic therapeutic formulation there is a requirement for a carrier system. The delivery of bacterial cells through the GIT is impaired by the harsh conditions of the upper GIT. Microencapsulation, specifically APA has been suggested as a method to overcome this obstacle [13]. This method is investigated in the presented research, specifically with relation to *L. fermentum* NCIMB 5221 and its FA producing capabilities. 

This work investigated the FA production of *L. fermentum* NCIMB 5221 in both the microencapsulated and the free form. The FAE activity of *L. fermentum* NCIMB 5221 free and encapsulated was determined by HPLC, to ensure that the microcapsule does not hamper the flow of substrate into and the FA product out of the microcapsule. Cell viability was determined during the assay to ensure that the cell count remained equal. Although FA production was non-significantly higher for free cells, this is explained by slightly higher free cell viability during the course of the experiment Figure 3. This is supported by the previous research that directly correlates bacterial cell counts of *L. fermentum* NCIMB 5221 with its FA production [12]. Keeping this in mind, the higher the cell count delivered to the colon, the higher the FA production. The successful delivery requires a carrier method such as microencapsulation.

Research has previously been presented on the use of microencapsulated cells for FA production, but fails to demonstrate a comparison between free and microencapsulated bacterial cells under the same conditions, as investigated in the presented research [38]. In terms of microencapsulation technology for the storage of microcapsules, Kailasapathy has demonstrated a significant increase in viability of microencapsulated cells in yoghurt cultures stored over 7 weeks, at a pH as low as 4 [39]. However, in terms of oral delivery, a pH of approximately 1.5 is encountered in the stomach. Hence, a microcapsule capable of delivering optimal numbers of bacteria through the GIT needs to sustain viability at such a low pH. In this flask study, the exposure to the simulated gastrointestinal conditions clearly illustrates the requirement for a carrier system when delivering live bacterial cells to the lower GIT Figure 5. A significant 2.5 log difference in viability following exposure to the simulated conditions could be detected between the free 1.10 × 10^4^ ± 1.00 × 10^3^ cfu/mL and the microencapsulated 5.50 × 10^6^ ± 1.00 × 10^5 ^*L. fermentum* NCIMB 5221.

Work is being undertaken to determine the FAE activity of free vs. microencapsulated *L. fermentum* NCIMB 5221 under GIT conditions. Unfortunately, the simple substrate EFA is labile under the enzymatic and pH conditions used in this study, which quickly resulted in EFA degradation when added to the SGF and SIF solutions. Normally, in the diet, the FA substrate would be present in a more complex form, such as wheat bran, which would permit FA release in the colon due to fermentation processes. This type of food matrix, however, is undefined, rendering it difficult to quantify the low levels of FA that are released. Our research continues to explore the enzymatic process, looking at other *in vitro* and, potentially, *in vivo* methods to comprehend the FAE activity in the GIT.

## 4. Conclusions

This work supports the use of APA microencapsulation for the oral delivery of the investigated probiotic. The presented work successfully demonstrated the advantage of using APA microencapsulation for *L. fermentum* NCIMB 5221 for use in oral delivery. The FAE enzymatic activity and bacterial viability were maintained post-encapsulation and the viability of encapsulated cells was greater than free cells in simulated gastrointestinal conditions. 

Future work should involve further optimisation of the microencapsulation process, since a significant loss of cell count was still evident with the microencapsulated formulation. Additional characterisation of a final formulation, in terms of the mechanisms of action and safety of the probiotic strain, the produced FA, and the use of the microencapsulation with *in vitro* and *in vivo* studies should be performed. In terms of the gastrointestinal tract, FAE activity should be characterized *in vivo* in appropriate animal models to ensure enzyme activity remains stable and efficient under potentially harsh conditions. The fate of the APA microcapsule should also be investigated *in vivo*. This work, nonetheless, opens up future potentials for the use of a synergistic formulation of microencapsulated *L. fermentu*m NCIMB 5221 with its intrinsic microbial FAE activity for both industrial and therapeutic applications. 
